# Bis(2,*S*-dimethyl­dithio­carbazate-κ^2^ *N* ^3^,*S*)(nitrato-κ*O*)copper(II) nitrate

**DOI:** 10.1107/S1600536812021423

**Published:** 2012-05-19

**Authors:** Saroj K. S. Hazari, B. K. Dey, B. Ganguly, Seik Weng Ng, Edward R. T. Tiekink

**Affiliations:** a Department of Chemistry, University of Chittagong, Chittagong 4331, Bangladesh; b Department of Chemistry, University of Malaya, 50603 Kuala Lumpur, Malaysia; c Chemistry Department, Faculty of Science, King Abdulaziz University, PO Box 80203 Jeddah, Saudi Arabia

## Abstract

The title complex, [Cu(NO_3_)(C_3_H_8_N_2_S_2_)_2_]NO_3_, represents a low-symmetry polymorph (*P*-1, *Z* = 4) of a previously reported form [*P*-1, *Z* = 2; Ali *et al.* (2011 ▶). *Polyhedron*, **30**, 542–548]. The Cu^II^ atom in each independent cation is found within a distorted square-pyramidal N_2_S_2_O coordination geometry defined by two *N*,*S*-bidentate ligands and an O atom derived from a monodentate nitrate. The primary difference between the cations is found in the relative orientations of the coordinated nitrate groups, which are directed to opposite sides of the mol­ecule. Supra­molecular layers along [110] and sustained by N—H⋯O inter­actions feature in the crystal packing. These are connected along the *c* axis by C—H⋯O inter­actions.

## Related literature

For related dithio­carbazate compounds, see: Hazari *et al.* (2012 ▶). For the previously reported polymorph, see: Ali *et al.* (2011 ▶). For additional structural analysis, see: Addison *et al.* (1984 ▶).
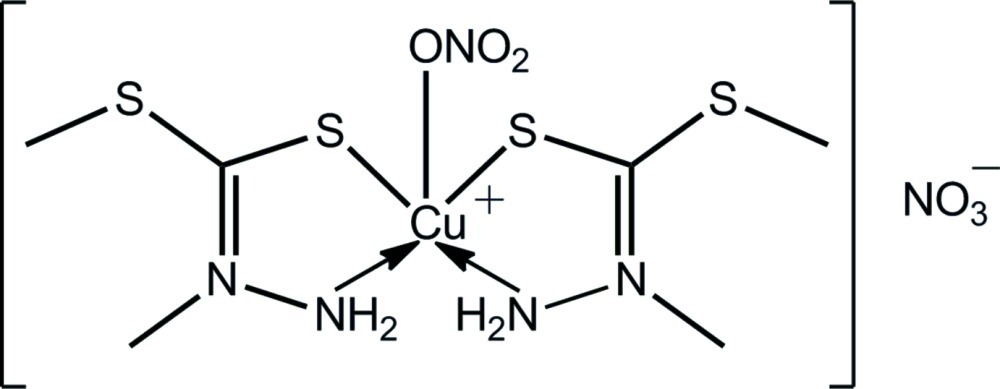


## Experimental

### 

#### Crystal data

[Cu(NO_3_)(C_3_H_8_N_2_S_2_)_2_]NO_3_
*M* *_r_* = 460.03
Triclinic, 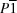

*a* = 11.2716 (4) Å
*b* = 12.1741 (4) Å
*c* = 13.8970 (5) Å
α = 115.449 (3)°
β = 100.734 (3)°
γ = 97.258 (3)°
*V* = 1645.39 (10) Å^3^
*Z* = 4
Mo *K*α radiation
μ = 1.87 mm^−1^
*T* = 100 K
0.40 × 0.20 × 0.10 mm

#### Data collection

Agilent SuperNova Dual diffractometer with an Atlas detector
Absorption correction: multi-scan (*CrysAlis PRO*; Agilent, 2011 ▶) *T*_min_ = 0.634, *T*_max_ = 1.000
25371 measured reflections
7555 independent reflections
5675 reflections with *I* > 2σ(*I*)
*R*_int_ = 0.080

#### Refinement

*R*[*F* ^2^ > 2σ(*F* ^2^)] = 0.059
*wR*(*F* ^2^) = 0.165
*S* = 1.09
7555 reflections
423 parameters
H-atom parameters constrained
Δρ_max_ = 1.15 e Å^−3^
Δρ_min_ = −0.95 e Å^−3^

### 

Data collection: *CrysAlis PRO* (Agilent, 2011 ▶); cell refinement: *CrysAlis PRO*; data reduction: *CrysAlis PRO*; program(s) used to solve structure: *SHELXS97* (Sheldrick, 2008 ▶); program(s) used to refine structure: *SHELXL97* (Sheldrick, 2008 ▶); molecular graphics: *ORTEP-3* (Farrugia, 1997 ▶), *DIAMOND* (Brandenburg, 2006 ▶) and *QMol* (Gans & Shalloway, 2001 ▶); software used to prepare material for publication: *publCIF* (Westrip, 2010 ▶).

## Supplementary Material

Crystal structure: contains datablock(s) global, I. DOI: 10.1107/S1600536812021423/hg5229sup1.cif

Structure factors: contains datablock(s) I. DOI: 10.1107/S1600536812021423/hg5229Isup2.hkl

Additional supplementary materials:  crystallographic information; 3D view; checkCIF report

## Figures and Tables

**Table 1:** Selected bond lengths (Å)

Cu1—S2	2.2502 (12)
Cu1—S3	2.2759 (12)
Cu1—O4	2.271 (3)
Cu1—N2	2.017 (4)
Cu1—N3	2.008 (4)
Cu2—S6	2.2724 (12)
Cu2—S7	2.2557 (12)
Cu2—O1	2.334 (3)
Cu2—N7	1.990 (4)
Cu2—N8	2.004 (4)

**Table 2:** Hydrogen-bond geometry (Å, °)

*D*—H⋯*A*	*D*—H	H⋯*A*	*D*⋯*A*	*D*—H⋯*A*
N2—H21⋯O5	0.88	2.21	2.860 (5)	131
N2—H22⋯O7	0.88	2.11	2.817 (5)	136
N3—H31⋯O3^i^	0.88	2.07	2.776 (5)	137
N3—H32⋯O11	0.88	2.05	2.881 (5)	156
N7—H71⋯O9	0.88	1.89	2.768 (5)	174
N7—H72⋯O6^ii^	0.88	2.10	2.807 (5)	137
N8—H81⋯O10	0.88	2.01	2.842 (5)	157
N8—H82⋯O2	0.88	2.08	2.894 (5)	154
C6—H6*A*⋯O8^iii^	0.98	2.47	3.295 (6)	141
C7—H7*A*⋯O12^iv^	0.98	2.48	3.247 (6)	135

Symmetry codes: (i) 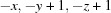; (ii) 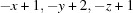; (iii) 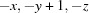; (iv) 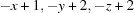.

**Fig. 1.**
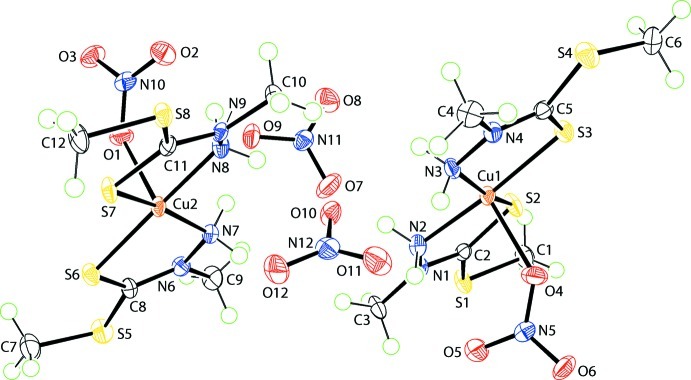
The molecular structures of the components comprising (I) showing the atom-labelling scheme and displacement ellipsoids at the 50% probability level.

**Fig. 2.**
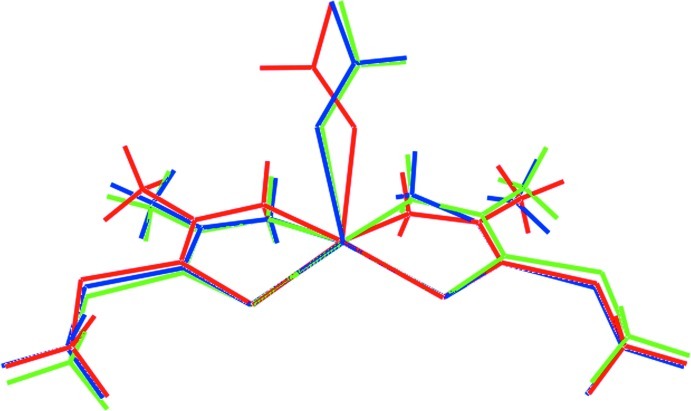
Overlay diagram of the two independent Cu-containing molecules comprising the asymmetric unit of (I). The first independent molecule (with the Cu1 atom) is shown in red. Also included is the molecule observed in the previously reported polymorph (green). The S—Cu—S residues in each molecule have been overlapped.

**Fig. 3.**
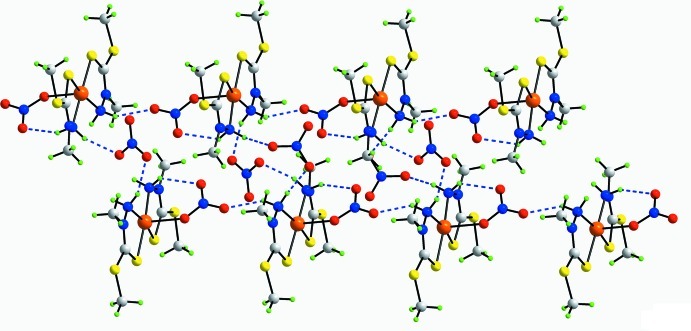
A view of the supramolecular chain along [110] in (I) mediated by N—H···O hydrogen bonding, shown as blue dashed lines.

**Fig. 4.**
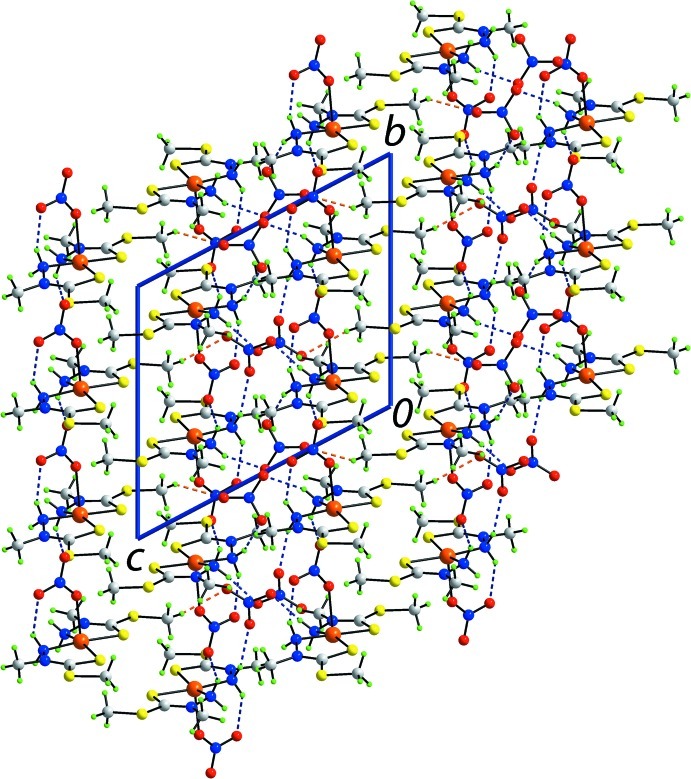
A view of the unit-cell contents in projection down the a axis in (I). The N—H···O and C—H···O interactions are shown as blue and orange dashed lines, respectively.

## supplementary crystallographic information

### Comment

As a continuation of systematic studies into the synthesis, characterization and biological activities of dithiocarbazates and their metal complexes (Hazari *et al.*, 2012), crystals of the title complex, (I), were isolated and characterized crystallographically.

Complex (I), Fig. 1, comprises two independent complex cations and two nitrate anions in the asymmetric unit, and represents a low symmetry (*P*1; *Z* = 4) polymorph of the previously reported triclinic form (*P*1, *Z* = 2; Ali *et al.*, 2011). The Cu^II^ atom is coordinated by a N_2_S_2_ donor set provided by two bidentate ligands and an O atom derived from a monodentate nitrate ligand, Table 1. The resulting N_2_S_2_O coordination geometry for the Cu1 atom is relatively close to a square pyramid as quantified by the value of τ = 0.15, which compares to the τ values of 0.0 and 1.0 for ideal square pyramidal and trigonal bipyramidal geometries, respectively (Addison *et al.*, 1984). The value for the Cu2 atom, *i.e.*τ = 0.22, indicates a small deviation along the path towards trigonal bipyramidal. The τ value for the previously described polymorph of 0.17 (Ali *et al.*, 2011) is intermediate between those calculated for the Cu atoms in (I). The primary difference between the cations comprising the asymmetric unit of (I) is found in the relative orientations of the coordinated nitrate groups, which are directed to opposite sides of the molecule. The orientation of the nitrate coordinated to the Cu2 atom matches that found in the literature polymorph. The three independent molecules are as illustrated in the overlay diagram, Fig. 2.

The crystal packing features significant hydrogen bonding interactions as expected from the composition. Thus, each amino-H atom forms a hydrogen bond to a nitrate-O atom, and each nitrate group atom participates in two N—H···O hydrogen bonds, Table 2. The result is the formation of a supramolecular layer along [110], Fig. 3. The layers are connected along the *c* axis by C–H···O interactions involving nitrate-O atoms not involved in N—H···O hydrogen bonds nor coordinated to a Cu centre, Fig. 4 and Table 2.

### Experimental

Copper(II) nitrate (1.17 g) was dissolved in dry ethanol (40 ml), in which a hot solution of 2,3-dimethyl-5-methylsulphanyl-[1,3,4]thiadiazolidine (2.4 g) in ethanol (40 ml) was added. The mixture was refluxed for 4 h. on a water bath. After reducing the volume and keeping overnight, a dark-blue product appeared, which was washed with ethanol (3 *x* 3 mL) and dried in a vacuum desiccator over silica gel. The product was recrystallized by dissoving the complex in ethanol (10 mL) and then layering this with petroleum ether (5 mL); *M*.pt: >493 K. The crystal structure determination showed that the original cyclic ligand had transformed to *N*-methyl-hydrazinecarbodithioic acid methyl ester (from which the cyclic form was prepared) during the course of the reaction.

### Refinement

The N– and C-bound H-atoms were placed in calculated positions (N—H = 0.88 Å and C—H = 0.98 Å) and were included in the refinement in the riding model approximation, with *U*_iso_(H) = 1.2*U*_equiv_(N) and 1.2*U*_equiv_(C). Owing to poor agreement, a number of reflections, *i.e.* (210 7), (1 11 0), (3 9 0), (12 0 6), (9 3 2), (7 7 3), (¯-10 2 7), (34 11) (12 0 12) and (84 11), were omitted from the final cycles of refinement. The maximum and minimum residual electron density peaks of 1.15 and 0.95 e Å^-3^, respectively, were located 0.87 Å and 1.17 Å from the Cu1 and N5 atoms, respectively.

### Crystal data

**Table:**

[Cu(NO_3_)(C_3_H_8_N_2_S_2_)_2_]NO_3_	*Z* = 4
*M**_r_* = 460.03	*F*(000) = 940
Triclinic, *P*1	*D*_x_ = 1.857 Mg m^−^^3^
Hall symbol: -P 1	Mo *K*α radiation, λ = 0.71073 Å
*a* = 11.2716 (4) Å	Cell parameters from 9482 reflections
*b* = 12.1741 (4) Å	θ = 2.3–27.5°
*c* = 13.8970 (5) Å	µ = 1.87 mm^−^^1^
α = 115.449 (3)°	*T* = 100 K
β = 100.734 (3)°	Prism, dark-blue
γ = 97.258 (3)°	0.40 × 0.20 × 0.10 mm
*V* = 1645.39 (10) Å^3^	

### Data collection

**Table:**

Agilent SuperNova Dual diffractometer with an Atlas detector	7555 independent reflections
Radiation source: SuperNova (Mo) X-ray Source	5675 reflections with *I* > 2σ(*I*)
Mirror monochromator	*R*_int_ = 0.080
Detector resolution: 10.4041 pixels mm^-1^	θ_max_ = 27.7°, θ_min_ = 2.7°
ω scan	*h* = −14→14
Absorption correction: multi-scan (*CrysAlis PRO*; Agilent, 2011)	*k* = −15→15
*T*_min_ = 0.634, *T*_max_ = 1.000	*l* = −18→18
25371 measured reflections	

### Refinement

**Table:**

Refinement on *F*^2^	Primary atom site location: structure-invariant direct methods
Least-squares matrix: full	Secondary atom site location: difference Fourier map
*R*[*F*^2^ > 2σ(*F*^2^)] = 0.059	Hydrogen site location: inferred from neighbouring sites
*wR*(*F*^2^) = 0.165	H-atom parameters constrained
*S* = 1.09	*w* = 1/[σ^2^(*F*_o_^2^) + (0.0751*P*)^2^ + 6.3265*P*] where *P* = (*F*_o_^2^ + 2*F*_c_^2^)/3
7555 reflections	(Δ/σ)_max_ = 0.001
423 parameters	Δρ_max_ = 1.15 e Å^−^^3^
0 restraints	Δρ_min_ = −0.95 e Å^−^^3^

### Special details

**Table:**

Geometry. All e.s.d.'s (except the e.s.d. in the dihedral angle between two l.s. planes) are estimated using the full covariance matrix. The cell e.s.d.'s are taken into account individually in the estimation of e.s.d.'s in distances, angles and torsion angles; correlations between e.s.d.'s in cell parameters are only used when they are defined by crystal symmetry. An approximate (isotropic) treatment of cell e.s.d.'s is used for estimating e.s.d.'s involving l.s. planes.
Refinement. Refinement of *F*^2^ against ALL reflections. The weighted *R*-factor *wR* and goodness of fit *S* are based on *F*^2^, conventional *R*-factors *R* are based on *F*, with *F* set to zero for negative *F*^2^. The threshold expression of *F*^2^ > σ(*F*^2^) is used only for calculating *R*-factors(gt) *etc*. and is not relevant to the choice of reflections for refinement. *R*-factors based on *F*^2^ are statistically about twice as large as those based on *F*, and *R*- factors based on ALL data will be even larger.

### Fractional atomic coordinates and isotropic or equivalent isotropic displacement parameters (Å^2^)

**Table:**

	*x*	*y*	*z*	*U*_iso_*/*U*_eq_	
Cu1	0.27022 (5)	0.71242 (5)	0.22467 (4)	0.01064 (15)	
Cu2	0.20705 (5)	0.78519 (5)	0.77159 (4)	0.01078 (15)	
S1	0.65195 (10)	0.59926 (12)	0.27050 (9)	0.0159 (2)	
S2	0.40155 (10)	0.59825 (11)	0.14944 (9)	0.0155 (2)	
S3	0.13598 (10)	0.65016 (11)	0.05685 (9)	0.0139 (2)	
S4	−0.11634 (11)	0.70264 (12)	0.01711 (10)	0.0188 (3)	
S5	0.60066 (11)	0.78742 (12)	0.95772 (10)	0.0190 (3)	
S6	0.35150 (10)	0.85031 (11)	0.93451 (9)	0.0143 (2)	
S7	0.06784 (10)	0.88609 (11)	0.84813 (9)	0.0139 (2)	
S8	−0.16110 (10)	0.92279 (11)	0.72725 (9)	0.0133 (2)	
O1	0.1587 (3)	0.5846 (3)	0.7551 (3)	0.0177 (7)	
O2	−0.0076 (3)	0.4891 (3)	0.6179 (3)	0.0199 (7)	
O3	0.0550 (3)	0.4046 (3)	0.7214 (3)	0.0202 (7)	
O4	0.3519 (3)	0.9027 (3)	0.2425 (3)	0.0232 (8)	
O5	0.5043 (3)	0.9861 (3)	0.3912 (3)	0.0235 (8)	
O6	0.4745 (3)	1.0785 (3)	0.2888 (3)	0.0203 (7)	
O7	0.3444 (3)	0.5699 (3)	0.4424 (3)	0.0263 (8)	
O8	0.2260 (3)	0.3833 (3)	0.3526 (3)	0.0233 (8)	
O9	0.2420 (4)	0.4953 (3)	0.5264 (3)	0.0245 (8)	
O10	0.2186 (3)	0.8161 (3)	0.5108 (3)	0.0165 (7)	
O11	0.2091 (4)	0.9896 (3)	0.5040 (3)	0.0255 (8)	
O12	0.2295 (3)	0.9840 (3)	0.6602 (3)	0.0254 (8)	
N1	0.4943 (3)	0.7112 (4)	0.3668 (3)	0.0106 (7)	
N2	0.3831 (3)	0.7541 (4)	0.3726 (3)	0.0116 (7)	
H21	0.4027	0.8358	0.4139	0.014*	
H22	0.3421	0.7212	0.4056	0.014*	
N3	0.1317 (3)	0.7749 (4)	0.2882 (3)	0.0127 (8)	
H31	0.1039	0.7274	0.3160	0.015*	
H32	0.1619	0.8512	0.3430	0.015*	
N4	0.0302 (3)	0.7770 (4)	0.2120 (3)	0.0114 (7)	
N5	0.4450 (3)	0.9892 (3)	0.3079 (3)	0.0119 (7)	
N6	0.4480 (3)	0.7287 (4)	0.7694 (3)	0.0130 (8)	
N7	0.3416 (3)	0.7342 (4)	0.6995 (3)	0.0124 (8)	
H71	0.3115	0.6601	0.6416	0.015*	
H72	0.3649	0.7874	0.6756	0.015*	
N8	0.0826 (3)	0.7273 (4)	0.6262 (3)	0.0123 (8)	
H81	0.1221	0.7324	0.5788	0.015*	
H82	0.0468	0.6480	0.6008	0.015*	
N9	−0.0108 (3)	0.7954 (3)	0.6308 (3)	0.0095 (7)	
N10	0.0668 (3)	0.4928 (4)	0.6978 (3)	0.0133 (8)	
N11	0.2703 (4)	0.4823 (4)	0.4388 (3)	0.0151 (8)	
N12	0.2198 (4)	0.9315 (4)	0.5595 (3)	0.0169 (8)	
C1	0.6459 (4)	0.5218 (5)	0.1266 (4)	0.0178 (10)	
H1A	0.7211	0.4904	0.1176	0.027*	
H1B	0.6405	0.5809	0.0963	0.027*	
H1C	0.5728	0.4516	0.0871	0.027*	
C2	0.5109 (4)	0.6429 (4)	0.2689 (4)	0.0110 (8)	
C3	0.5857 (4)	0.7512 (5)	0.4715 (4)	0.0152 (9)	
H3A	0.6280	0.6840	0.4663	0.023*	
H3B	0.5440	0.7704	0.5307	0.023*	
H3C	0.6468	0.8260	0.4879	0.023*	
C4	−0.0671 (4)	0.8334 (5)	0.2569 (4)	0.0176 (10)	
H4A	−0.1007	0.8761	0.2159	0.026*	
H4B	−0.0321	0.8940	0.3352	0.026*	
H4C	−0.1337	0.7680	0.2496	0.026*	
C5	0.0214 (4)	0.7144 (4)	0.1053 (4)	0.0119 (9)	
C6	−0.0918 (5)	0.6233 (5)	−0.1177 (4)	0.0203 (10)	
H6A	−0.1655	0.6124	−0.1740	0.030*	
H6B	−0.0769	0.5412	−0.1303	0.030*	
H6C	−0.0196	0.6730	−0.1223	0.030*	
C7	0.5800 (5)	0.8552 (5)	1.0945 (4)	0.0224 (11)	
H7A	0.6548	0.8614	1.1473	0.034*	
H7B	0.5086	0.8024	1.0969	0.034*	
H7C	0.5653	0.9389	1.1141	0.034*	
C8	0.4610 (4)	0.7851 (4)	0.8777 (4)	0.0133 (9)	
C9	0.5417 (4)	0.6735 (5)	0.7175 (4)	0.0170 (10)	
H9A	0.5769	0.6266	0.7534	0.025*	
H9B	0.6078	0.7400	0.7257	0.025*	
H9C	0.5032	0.6169	0.6388	0.025*	
C10	−0.0875 (4)	0.7751 (4)	0.5256 (4)	0.0139 (9)	
H10A	−0.1248	0.8467	0.5369	0.021*	
H10B	−0.1533	0.6989	0.4947	0.021*	
H10C	−0.0362	0.7660	0.4741	0.021*	
C11	−0.0305 (4)	0.8604 (4)	0.7285 (4)	0.0113 (8)	
C12	−0.1614 (4)	0.9903 (5)	0.8701 (4)	0.0174 (10)	
H12A	−0.2341	1.0262	0.8793	0.026*	
H12B	−0.0856	1.0562	0.9150	0.026*	
H12C	−0.1646	0.9254	0.8941	0.026*	

### Atomic displacement parameters (Å^2^)

**Table:**

	*U*^11^	*U*^22^	*U*^33^	*U*^12^	*U*^13^	*U*^23^
Cu1	0.0092 (3)	0.0148 (3)	0.0094 (3)	0.0038 (2)	0.0040 (2)	0.0061 (2)
Cu2	0.0107 (3)	0.0145 (3)	0.0087 (3)	0.0045 (2)	0.0051 (2)	0.0053 (2)
S1	0.0118 (5)	0.0270 (6)	0.0099 (5)	0.0089 (5)	0.0055 (4)	0.0072 (5)
S2	0.0147 (5)	0.0221 (6)	0.0080 (5)	0.0077 (5)	0.0028 (4)	0.0047 (5)
S3	0.0117 (5)	0.0191 (6)	0.0109 (5)	0.0048 (4)	0.0040 (4)	0.0062 (4)
S4	0.0141 (5)	0.0261 (7)	0.0169 (6)	0.0074 (5)	0.0027 (4)	0.0105 (5)
S5	0.0137 (5)	0.0261 (7)	0.0180 (6)	0.0077 (5)	0.0043 (5)	0.0101 (5)
S6	0.0119 (5)	0.0195 (6)	0.0100 (5)	0.0049 (4)	0.0045 (4)	0.0046 (4)
S7	0.0154 (5)	0.0204 (6)	0.0079 (5)	0.0079 (4)	0.0055 (4)	0.0063 (4)
S8	0.0124 (5)	0.0191 (6)	0.0102 (5)	0.0066 (4)	0.0055 (4)	0.0066 (4)
O1	0.0163 (16)	0.0150 (17)	0.0197 (17)	−0.0026 (13)	0.0004 (13)	0.0095 (14)
O2	0.0180 (17)	0.0208 (18)	0.0191 (18)	0.0047 (14)	0.0013 (14)	0.0089 (15)
O3	0.0219 (17)	0.0162 (17)	0.0255 (19)	0.0010 (14)	0.0068 (15)	0.0131 (15)
O4	0.0195 (17)	0.0203 (18)	0.0231 (19)	−0.0062 (14)	−0.0062 (15)	0.0119 (16)
O5	0.0216 (18)	0.026 (2)	0.0210 (19)	−0.0025 (15)	−0.0026 (15)	0.0142 (16)
O6	0.0257 (18)	0.0164 (17)	0.0232 (19)	0.0010 (14)	0.0095 (15)	0.0130 (15)
O7	0.029 (2)	0.023 (2)	0.031 (2)	−0.0001 (16)	0.0144 (17)	0.0157 (17)
O8	0.0241 (18)	0.0227 (19)	0.0165 (18)	0.0043 (15)	0.0075 (15)	0.0026 (15)
O9	0.034 (2)	0.0234 (19)	0.0134 (17)	−0.0020 (16)	0.0096 (15)	0.0076 (15)
O10	0.0196 (17)	0.0166 (17)	0.0159 (16)	0.0060 (13)	0.0114 (14)	0.0067 (14)
O11	0.032 (2)	0.0192 (19)	0.0225 (19)	0.0047 (16)	−0.0014 (16)	0.0106 (16)
O12	0.028 (2)	0.027 (2)	0.0144 (18)	0.0047 (16)	0.0112 (15)	0.0020 (15)
N1	0.0094 (17)	0.0160 (19)	0.0099 (18)	0.0053 (14)	0.0047 (14)	0.0076 (15)
N2	0.0140 (18)	0.0132 (19)	0.0108 (18)	0.0082 (15)	0.0069 (15)	0.0056 (15)
N3	0.0112 (17)	0.018 (2)	0.0090 (18)	0.0026 (15)	0.0010 (14)	0.0073 (16)
N4	0.0088 (17)	0.0159 (19)	0.0117 (18)	0.0047 (14)	0.0042 (14)	0.0073 (16)
N5	0.0113 (17)	0.0116 (18)	0.0150 (19)	0.0051 (14)	0.0087 (15)	0.0053 (15)
N6	0.0101 (17)	0.016 (2)	0.0129 (19)	0.0040 (15)	0.0057 (15)	0.0056 (16)
N7	0.0132 (18)	0.0137 (19)	0.0103 (18)	0.0019 (15)	0.0042 (15)	0.0055 (15)
N8	0.0140 (18)	0.0161 (19)	0.0113 (18)	0.0089 (15)	0.0067 (15)	0.0077 (16)
N9	0.0089 (16)	0.0122 (18)	0.0098 (17)	0.0034 (14)	0.0051 (14)	0.0061 (15)
N10	0.0101 (17)	0.0131 (19)	0.017 (2)	0.0048 (15)	0.0083 (15)	0.0047 (16)
N11	0.0132 (18)	0.018 (2)	0.018 (2)	0.0046 (15)	0.0061 (16)	0.0099 (17)
N12	0.0109 (18)	0.021 (2)	0.018 (2)	0.0034 (16)	0.0054 (15)	0.0072 (17)
C1	0.016 (2)	0.025 (3)	0.012 (2)	0.0083 (19)	0.0065 (18)	0.006 (2)
C2	0.013 (2)	0.009 (2)	0.010 (2)	0.0026 (16)	0.0056 (16)	0.0036 (17)
C3	0.012 (2)	0.025 (3)	0.008 (2)	0.0068 (18)	0.0028 (17)	0.0054 (19)
C4	0.015 (2)	0.019 (2)	0.019 (2)	0.0091 (19)	0.0097 (19)	0.006 (2)
C5	0.010 (2)	0.013 (2)	0.014 (2)	0.0021 (16)	0.0036 (17)	0.0075 (18)
C6	0.024 (2)	0.025 (3)	0.017 (2)	0.005 (2)	0.006 (2)	0.014 (2)
C7	0.022 (2)	0.030 (3)	0.015 (2)	0.007 (2)	0.004 (2)	0.011 (2)
C8	0.015 (2)	0.014 (2)	0.009 (2)	0.0033 (17)	0.0047 (17)	0.0039 (18)
C9	0.015 (2)	0.021 (2)	0.016 (2)	0.0094 (19)	0.0099 (18)	0.0060 (19)
C10	0.014 (2)	0.019 (2)	0.010 (2)	0.0056 (18)	0.0025 (17)	0.0072 (18)
C11	0.011 (2)	0.014 (2)	0.011 (2)	0.0032 (17)	0.0059 (16)	0.0063 (18)
C12	0.017 (2)	0.025 (3)	0.010 (2)	0.010 (2)	0.0076 (18)	0.0050 (19)

### Geometric parameters (Å, º)

**Table:**

Cu1—S2	2.2502 (12)	N3—N4	1.417 (5)
Cu1—S3	2.2759 (12)	N3—H31	0.8800
Cu1—O4	2.271 (3)	N3—H32	0.8800
Cu1—N2	2.017 (4)	N4—C5	1.321 (6)
Cu1—N3	2.008 (4)	N4—C4	1.463 (6)
Cu2—S6	2.2724 (12)	N6—C8	1.328 (6)
Cu2—S7	2.2557 (12)	N6—N7	1.424 (5)
Cu2—O1	2.334 (3)	N6—C9	1.459 (6)
Cu2—N7	1.990 (4)	N7—H71	0.8800
Cu2—N8	2.004 (4)	N7—H72	0.8800
S1—C2	1.738 (4)	N8—N9	1.414 (5)
S1—C1	1.790 (5)	N8—H81	0.8800
S2—C2	1.692 (5)	N8—H82	0.8800
S3—C5	1.691 (4)	N9—C11	1.324 (6)
S4—C5	1.737 (4)	N9—C10	1.454 (6)
S4—C6	1.795 (5)	C1—H1A	0.9800
S5—C8	1.739 (5)	C1—H1B	0.9800
S5—C7	1.793 (5)	C1—H1C	0.9800
S6—C8	1.688 (5)	C3—H3A	0.9800
S7—C11	1.694 (5)	C3—H3B	0.9800
S8—C11	1.740 (4)	C3—H3C	0.9800
S8—C12	1.794 (5)	C4—H4A	0.9800
O1—N10	1.262 (5)	C4—H4B	0.9800
O2—N10	1.241 (5)	C4—H4C	0.9800
O3—N10	1.250 (5)	C6—H6A	0.9800
O4—N5	1.257 (5)	C6—H6B	0.9800
O5—N5	1.242 (5)	C6—H6C	0.9800
O6—N5	1.246 (5)	C7—H7A	0.9800
O7—N11	1.246 (5)	C7—H7B	0.9800
O8—N11	1.234 (5)	C7—H7C	0.9800
O9—N11	1.264 (5)	C9—H9A	0.9800
O10—N12	1.268 (5)	C9—H9B	0.9800
O11—N12	1.252 (5)	C9—H9C	0.9800
O12—N12	1.239 (5)	C10—H10A	0.9800
N1—C2	1.318 (6)	C10—H10B	0.9800
N1—N2	1.420 (5)	C10—H10C	0.9800
N1—C3	1.457 (6)	C12—H12A	0.9800
N2—H21	0.8800	C12—H12B	0.9800
N2—H22	0.8800	C12—H12C	0.9800
			
N3—Cu1—N2	94.04 (14)	O2—N10—O3	121.1 (4)
N3—Cu1—S2	166.11 (12)	O2—N10—O1	120.4 (4)
N2—Cu1—S2	86.27 (11)	O3—N10—O1	118.4 (4)
N3—Cu1—O4	91.59 (15)	O8—N11—O7	121.4 (4)
N2—Cu1—O4	91.09 (14)	O8—N11—O9	119.8 (4)
S2—Cu1—O4	102.29 (11)	O7—N11—O9	118.8 (4)
N3—Cu1—S3	85.68 (11)	O12—N12—O11	121.4 (4)
N2—Cu1—S3	175.33 (12)	O12—N12—O10	119.7 (4)
S2—Cu1—S3	92.88 (4)	O11—N12—O10	118.9 (4)
O4—Cu1—S3	93.58 (9)	S1—C1—H1A	109.5
N7—Cu2—N8	92.53 (15)	S1—C1—H1B	109.5
N7—Cu2—S7	165.60 (12)	H1A—C1—H1B	109.5
N8—Cu2—S7	85.68 (11)	S1—C1—H1C	109.5
N7—Cu2—S6	86.25 (11)	H1A—C1—H1C	109.5
N8—Cu2—S6	178.76 (11)	H1B—C1—H1C	109.5
S7—Cu2—S6	95.44 (4)	N1—C2—S2	122.5 (3)
N7—Cu2—O1	87.60 (14)	N1—C2—S1	115.5 (3)
N8—Cu2—O1	89.83 (14)	S2—C2—S1	122.0 (3)
S7—Cu2—O1	106.66 (9)	N1—C3—H3A	109.5
S6—Cu2—O1	90.35 (9)	N1—C3—H3B	109.5
C2—S1—C1	102.6 (2)	H3A—C3—H3B	109.5
C2—S2—Cu1	97.03 (15)	N1—C3—H3C	109.5
C5—S3—Cu1	96.68 (16)	H3A—C3—H3C	109.5
C5—S4—C6	103.3 (2)	H3B—C3—H3C	109.5
C8—S5—C7	102.6 (2)	N4—C4—H4A	109.5
C8—S6—Cu2	95.88 (16)	N4—C4—H4B	109.5
C11—S7—Cu2	96.78 (15)	H4A—C4—H4B	109.5
C11—S8—C12	102.0 (2)	N4—C4—H4C	109.5
N10—O1—Cu2	133.0 (3)	H4A—C4—H4C	109.5
N5—O4—Cu1	133.9 (3)	H4B—C4—H4C	109.5
C2—N1—N2	119.0 (4)	N4—C5—S3	122.7 (3)
C2—N1—C3	124.2 (4)	N4—C5—S4	115.4 (3)
N2—N1—C3	116.7 (3)	S3—C5—S4	121.8 (3)
N1—N2—Cu1	114.4 (3)	S4—C6—H6A	109.5
N1—N2—H21	108.6	S4—C6—H6B	109.5
Cu1—N2—H21	108.6	H6A—C6—H6B	109.5
N1—N2—H22	108.6	S4—C6—H6C	109.5
Cu1—N2—H22	108.6	H6A—C6—H6C	109.5
H21—N2—H22	107.6	H6B—C6—H6C	109.5
N4—N3—Cu1	115.0 (3)	S5—C7—H7A	109.5
N4—N3—H31	108.5	S5—C7—H7B	109.5
Cu1—N3—H31	108.5	H7A—C7—H7B	109.5
N4—N3—H32	108.5	S5—C7—H7C	109.5
Cu1—N3—H32	108.5	H7A—C7—H7C	109.5
H31—N3—H32	107.5	H7B—C7—H7C	109.5
C5—N4—N3	118.3 (4)	N6—C8—S6	123.1 (3)
C5—N4—C4	124.2 (4)	N6—C8—S5	114.8 (3)
N3—N4—C4	116.9 (4)	S6—C8—S5	122.1 (3)
O5—N5—O6	121.1 (4)	N6—C9—H9A	109.5
O5—N5—O4	120.0 (4)	N6—C9—H9B	109.5
O6—N5—O4	118.9 (4)	H9A—C9—H9B	109.5
C8—N6—N7	117.8 (4)	N6—C9—H9C	109.5
C8—N6—C9	124.5 (4)	H9A—C9—H9C	109.5
N7—N6—C9	117.3 (4)	H9B—C9—H9C	109.5
N6—N7—Cu2	114.5 (3)	N9—C10—H10A	109.5
N6—N7—H71	108.6	N9—C10—H10B	109.5
Cu2—N7—H71	108.6	H10A—C10—H10B	109.5
N6—N7—H72	108.6	N9—C10—H10C	109.5
Cu2—N7—H72	108.6	H10A—C10—H10C	109.5
H71—N7—H72	107.6	H10B—C10—H10C	109.5
N9—N8—Cu2	114.5 (3)	N9—C11—S7	122.3 (3)
N9—N8—H81	108.6	N9—C11—S8	115.9 (3)
Cu2—N8—H81	108.6	S7—C11—S8	121.8 (3)
N9—N8—H82	108.6	S8—C12—H12A	109.5
Cu2—N8—H82	108.6	S8—C12—H12B	109.5
H81—N8—H82	107.6	H12A—C12—H12B	109.5
C11—N9—N8	117.6 (4)	S8—C12—H12C	109.5
C11—N9—C10	125.2 (4)	H12A—C12—H12C	109.5
N8—N9—C10	116.6 (3)	H12B—C12—H12C	109.5
			
N3—Cu1—S2—C2	−98.7 (5)	S6—Cu2—N7—N6	15.0 (3)
N2—Cu1—S2—C2	−6.90 (19)	O1—Cu2—N7—N6	−75.5 (3)
O4—Cu1—S2—C2	83.40 (18)	N7—Cu2—N8—N9	−149.6 (3)
S3—Cu1—S2—C2	177.70 (16)	S7—Cu2—N8—N9	16.1 (3)
N3—Cu1—S3—C5	5.78 (19)	S6—Cu2—N8—N9	−139 (5)
N2—Cu1—S3—C5	92.5 (13)	O1—Cu2—N8—N9	122.8 (3)
S2—Cu1—S3—C5	171.94 (16)	Cu2—N8—N9—C11	−20.0 (5)
O4—Cu1—S3—C5	−85.54 (18)	Cu2—N8—N9—C10	167.7 (3)
N7—Cu2—S6—C8	−9.96 (19)	Cu2—O1—N10—O2	15.7 (6)
N8—Cu2—S6—C8	−21 (5)	Cu2—O1—N10—O3	−166.4 (3)
S7—Cu2—S6—C8	−175.62 (16)	N2—N1—C2—S2	−3.0 (6)
O1—Cu2—S6—C8	77.61 (18)	C3—N1—C2—S2	179.8 (3)
N7—Cu2—S7—C11	74.6 (5)	N2—N1—C2—S1	178.0 (3)
N8—Cu2—S7—C11	−8.69 (19)	C3—N1—C2—S1	0.7 (6)
S6—Cu2—S7—C11	170.78 (16)	Cu1—S2—C2—N1	7.4 (4)
O1—Cu2—S7—C11	−97.22 (18)	Cu1—S2—C2—S1	−173.6 (2)
N7—Cu2—O1—N10	−111.8 (4)	C1—S1—C2—N1	−175.2 (4)
N8—Cu2—O1—N10	−19.2 (4)	C1—S1—C2—S2	5.8 (3)
S7—Cu2—O1—N10	66.2 (4)	N3—N4—C5—S3	−9.1 (6)
S6—Cu2—O1—N10	162.0 (4)	C4—N4—C5—S3	−179.8 (3)
N3—Cu1—O4—N5	105.5 (4)	N3—N4—C5—S4	171.0 (3)
N2—Cu1—O4—N5	11.4 (4)	C4—N4—C5—S4	0.4 (6)
S2—Cu1—O4—N5	−75.0 (4)	Cu1—S3—C5—N4	−0.1 (4)
S3—Cu1—O4—N5	−168.7 (4)	Cu1—S3—C5—S4	179.7 (2)
C2—N1—N2—Cu1	−4.4 (5)	C6—S4—C5—N4	176.2 (3)
C3—N1—N2—Cu1	173.1 (3)	C6—S4—C5—S3	−3.7 (4)
N3—Cu1—N2—N1	173.2 (3)	N7—N6—C8—S6	5.9 (6)
S2—Cu1—N2—N1	7.1 (3)	C9—N6—C8—S6	178.8 (4)
O4—Cu1—N2—N1	−95.2 (3)	N7—N6—C8—S5	−173.9 (3)
S3—Cu1—N2—N1	86.8 (14)	C9—N6—C8—S5	−1.0 (6)
N2—Cu1—N3—N4	173.4 (3)	Cu2—S6—C8—N6	5.2 (4)
S2—Cu1—N3—N4	−95.8 (5)	Cu2—S6—C8—S5	−175.0 (3)
O4—Cu1—N3—N4	82.2 (3)	C7—S5—C8—N6	−174.7 (4)
S3—Cu1—N3—N4	−11.3 (3)	C7—S5—C8—S6	5.5 (4)
Cu1—N3—N4—C5	14.6 (5)	N8—N9—C11—S7	11.7 (6)
Cu1—N3—N4—C4	−174.1 (3)	C10—N9—C11—S7	−176.7 (3)
Cu1—O4—N5—O5	−9.9 (6)	N8—N9—C11—S8	−170.5 (3)
Cu1—O4—N5—O6	171.7 (3)	C10—N9—C11—S8	1.1 (6)
C8—N6—N7—Cu2	−15.9 (5)	Cu2—S7—C11—N9	1.2 (4)
C9—N6—N7—Cu2	170.7 (3)	Cu2—S7—C11—S8	−176.6 (2)
N8—Cu2—N7—N6	−165.2 (3)	C12—S8—C11—N9	175.4 (3)
S7—Cu2—N7—N6	112.3 (5)	C12—S8—C11—S7	−6.7 (3)

### Hydrogen-bond geometry (Å, º)

**Table:**

*D*—H···*A*	*D*—H	H···*A*	*D*···*A*	*D*—H···*A*
N2—H21···O5	0.88	2.21	2.860 (5)	131
N2—H22···O7	0.88	2.11	2.817 (5)	136
N3—H31···O3^i^	0.88	2.07	2.776 (5)	137
N3—H32···O11	0.88	2.05	2.881 (5)	156
N7—H71···O9	0.88	1.89	2.768 (5)	174
N7—H72···O6^ii^	0.88	2.10	2.807 (5)	137
N8—H81···O10	0.88	2.01	2.842 (5)	157
N8—H82···O2	0.88	2.08	2.894 (5)	154
C6—H6*A*···O8^iii^	0.98	2.47	3.295 (6)	141
C7—H7*A*···O12^iv^	0.98	2.48	3.247 (6)	135

Symmetry codes: (i) −*x*, −*y*+1, −*z*+1; (ii) −*x*+1, −*y*+2, −*z*+1; (iii) −*x*, −*y*+1, −*z*; (iv) −*x*+1, −*y*+2, −*z*+2.
